# PolyPIMuse significantly increases fall risk: comparison of three medication evaluation tools

**DOI:** 10.3389/fragi.2026.1903105

**Published:** 2026-08-21

**Authors:** András Érszegi, Réka Viola, Bettina Vargáné Szabó, Zsófia Engi, Mária Matuz, Ria Benkő, Zoltán Pető, Dezső Csupor

**Affiliations:** 1 Faculty of Pharmacy, Institute of Clinical Pharmacy, University of Szeged, Szeged, Hungary; 2 Albert Szent-Györgyi Health Center, Institute of Clinical PharmacyUniversity of Szeged, Szeged, Hungary; 3 Albert Szent-Györgyi Health Center, Department of Emergency MedicineUniversity of Szeged, Szeged, Hungary; 4 Institute for Translational Medicine, University of Pécs, Pécs, Hungary

**Keywords:** elderly, EU(7)PIM, falls, FORTA, Hungary, PIM list, PRISCUS

## Abstract

**Introduction:**

Falls are a leading cause of injury and hospitalization in older adults, with pharmacotherapy identified as a modifiable risk factor. Tools for identifying potentially inappropriate medications (PIMs), such as the EU(7)PIM, PRISCUS, and FORTA lists, differ in methodology. However, their comparative performance in predicting fall risk has not been systematically evaluated. The aim of our work was to investigate the association between PIM use and fall risk among older adults and to analyze the correlations between PIM utilization based on three PIM lists (EU(7)PIM, PRISCUS, and FORTA C/D) and fall risk.

**Methods:**

In a retrospective case -control study based on real-world data from Hungarian emergency and primary care settings, we analyzed fall-related emergency department visits (n=886) in individuals aged ≥65 years and matched them with a control group from general practices (n = 1364).

**Results:**

Use of PIMs identified by all three lists was significantly associated with increased fall risk. (Use of 1 FORTA C/D active substance: AOR: 1.434; CI95%:1.120–1.838; p = 0.004; Use of 1 EU(7)PIM active substance: AOR: 1.201; CI95%: 0.907–1.591; p = 0.200; Use of 1 PRISCUS active substance: AOR: 1.390; CI95%:1.069–1.808; p = 0.014) Of the three lists, use of drugs on the PRISCUS list was most significantly associated with increased fall risk. (Use of 2 PRISCUS active substance: AOR: 1.557; CI95%:1.148–2.114; p = 0.004; Use of 3 PRISCUS active substance: AOR: 2.045; CI95%: 1.408–2.982; p = 0.000; Use of 4 PRISCUS active substance: AOR: 1.869; CI95%: 1.149–3.065; p = 0.012; Use of 5 PRISCUS active substance: AOR: 2.638; CI95%: 1.464–4.895; p = 0.002).

**Discussion:**

We introduce the concept of “polyPIMuse” –the use of ≥5 PIM-listed drugs –which further elevated fall risk, particularly for PRISCUS and FORTA C/D lists. Despite methodological differences, all three PIM tools proved useful in predicting fall risk. Incorporating these tools into routine medication reviews may aid fall prevention strategies in geriatric care.

## Introduction

The frequency of falls increases with age, and this risk is influenced by several lifestyle and other factors, including medication (Thapa and others et al., 1996; Nevitt et al., 1991; Tinetti, 2003; Rubenstein and Josephson, 2002). In 2020, 27% of adults aged ≥65 years in the United States reported experiencing at least one fall in the previous year (Ramakrishna kakara and others, 2023).

Falls frequently cause injury, typically minor soft-tissue injuries, such as bruises and scrapes. In a 2-year study that surveyed women aged >70 years, 41% of falls resulted in minor injuries, while 6% caused major ones (Nancy, 2007). A previous study found that 5%–10% of falls among older adults living in communities resulted in major injuries, such as fractures, head trauma, or significant lacerations (Rubenstein and Josephson, 2006). The incidence of fall-related major injuries is higher among nursing home residents at 10%–30% (Oliver and others et al., 2007). Falls accounted for 62% of nonfatal injuries requiring visits to U.S. emergency departments among people aged >65 years (Public Health and Aging, 2003). Furthermore, approximately 5% of falls experienced by older people require hospitalization (Society AG, Society G, Of AA, On Falls Prevention OSP., 2001).

As populations age, the number of hospitalizations due to falls is expected to increase significantly. In the Netherlands, the number of fall-related hospitalizations more than doubled between 1981 and 2008, with a lower average length of stay, partially offset by the total number of days spent in hospitals due to falls (Klaas, 2010). In the United States, fall-related injuries are a significant cause of hospital readmissions among older adults, especially for individuals with a previous hospitalization history due to a fall-related injury and those with cognitive impairments (Geoffrey, 2019).

Numerous community-based prospective cohort studies have been conducted to investigate the risk factors of falls (Tinetti et al., 1988) (Graafmans and others et al., 1996) (Campbell et al., 1989) (Ho et al., 1996) (Davis and others et al., 1999) (Schwartz and o et al., 1999). The risk factors identified in at least two of these studies include a history of falls, lower extremity weakness, advancing age, female sex, cognitive impairment, balance problems, use of psychotropic drugs, arthritis, previous stroke, orthostatic hypotension, dizziness, and anemia. Furthermore, multiple studies have associated falls with syncope, a history of previous falls resulting in injury, and decreased executive function as measured by the Trail Making Test B time (Schiffer et al., 2003).

A systematic review and meta-analysis explored various drug groups and concluded that nonsteroidal anti-inflammatory drugs, opioids, loop diuretics, benzodiazepines, antidepressants, and antipsychotics could increase the risk of falls, consequently categorizing them as Falling Risk Increasing Drugs (Max de Vries and others, 2018) (Lotta, 2018a) (Lotta, 2018b). Many tools and lists, called Potentially Inappropriate Medication (PIM) lists, have been developed to assess the medications of elderly patients. These lists can differ from one another, are explicit or implicit, According to the latest approach, these are patient- or drug-oriented. Examples include DOLA (Drug-Oriented Listing Approach), DOLA+ (DOLA with disease specification), or PILA (Patient In-focus Listing Approach) (Pazan et al., 2019). DOLAs (e.g., Beers list; EU (7) PIM list; DOLA+: PRISCUS list) are easier to use because the only required dataset is the elderly patient medication list. However, for PILAs (e.g., FORTA (Fit fOR The Aged) list) specific patient information (e.g., drug indication, morbidities) is required. Previous studies have found an association between PIM use and an increased risk of falling (Érszegi and others et al., 2024). However, the use of these tools and lists in daily practice has not been investigated. Falls carry a risk of serious injuries and associated treatment costs, which impose a burden on the patient, their family, and the healthcare system. Thus, the impact of medication on fall risk must be evaluated.

## Objective

We compared the utilization of the FORTA, PRISCUS (latin for old and venerable), and European Union list of potentially inappropriate medications (EU (7) PIM) lists to analyze the correlations between PIM utilization based on each list and fall risk. Furthermore, we compared the lists to determine the list that would best demonstrate the influence of medications on the risk of falling.

## Methods

### Study design

Our case–control study was designed and conducted according to the STROBE guidelines. The study was based on patient data collected by the eMedSol Hospital Information System (HIS) between 21.03.2022 and 06.07.2022 on fall incidence among older people aged ≥65 years who visited the Department of Emergency Medicine at the Albert Szent-Györgyi Health Centre of the University of Szeged (ED). The study period, including the start and end dates of data collection, was determined by consensus among three authors (András Érszegi, Réka Viola, and Dezső Csupor). The falls were grouped by time, type of fall, and injuries sustained as categorized using codes in the 10^th^ revision of the International Statistical Classification of Diseases and Related Health Problems (ICD-10). Available laboratory parameters were also collected. Data from three general practice clinics in Hungary, two in Szeged and one in Szatymaz, were used as control groups. In this study, only chronically used drugs, which used every day by the patient (not as needed) (active substance name, dosage and administration were recorded) were collected and evaluated. Moreover, the number of medications and drugs on the PIM lists (1–5) used were recorded. Patient data were collected, including age, sex, Alzheimer’s disease status, Parkinson’s, dementia status (as important morbidities as falling risk factors), and polypharmacy.

### PRISCUS list

When the use of PIM cannot be avoided, the PRISCUS list offers guidance on treatment options and recommended steps. This list was formulated through a structured expert survey conducted in a two-round Delphi process. It contains 177 active substances and is considered a DOLA+ (Holt et al., 2010) (Mann et al., 2023).

### EU (7)PIM list

The EU (7)PIM list was established in 2015 and includes various national PIM lists, including the German PRISCUS list and PIM lists used in France and Canada. It contains 282 medications and 34 drug categories, provides data on drug dosage and duration limitations, and lists therapeutic alternatives and guidance on dosage adjustments. This list was developed through two rounds of Delphi surveys conducted with the participation of 30 experts (Renom-G et al., 2015).

### Modified FORTA list

The FORTA list is considered a PILA. It classifies drugs into groups A to D based on whether they are appropriate in the used indication in older patients (Pazan et al., 2022). Classification into the A/B group indicates proper usage, whereas that in C/D mean inappropriate indication. Therefore, our study focused only on the C/D list, which included 88 active substances. Some active substances are rated A/B in one indication but rated C/D in another. In this study, we did not have information on the comorbidities of the patients; therefore, we included FORTA C/D rated PIM drugs in the analysis.

### Patients

The study group consisted of people aged ≥65 years who were diagnosed with ICD-10 codes S or W00-W19 in the ED after a fall in the catchment area. The control group consisted of people aged ≥65 years who have not experienced a fall.

### Medications

Data on patient medical history were collected from the HIS. If the patient’s medical history was not documented, telephone calls were made to collect information about their prescribed medications. Patients were excluded if they could not be reached after three attempts. Only data on prescribed medications were collected because over-the-counter drugs are not recorded in medical systems. Drug name, then converted to active substance name (The official Hungarian Medicine Register database was used: https://ogyei.gov.hu/gyogyszeradatbazis), dosage and administration time (at night, in the morning, two times a day, etc.) were collected. The drugs used were classified using the World Health Organization Collaborative Centre for Drug Statistics Methodology (ATC code system). Polypharmacy was defined as the use of five or more active substances.

### Statistical analysis

Statistical analysis was performed using RStudio software (version 2024.12.0) using the *MatchIt* and *glmnet* packages. A two-step modeling process was employed: First, a generalized linear model (GLM) with a binomial link function was used to explore the relationship between the target variable (fall risk) and feature variables (e.g., use of PIM drugs) on the full dataset. Because age, sex, and presence of neurodegenerative disease (Parkinson’s, Alzheimer’s, dementia) were not equally represented across the two classes of the target variable, an adjusted subset was created using the *MatchIt* package. A second GLM, using only the derived drug-related variables as independent predictors, was fitted on this adjusted subset to isolate their effects. Statistical significance was set at α = 0.05, and 95% confidence intervals (CI) were reported for all adjusted odds ratios (AOR). Model evaluation was performed using McFadden’s R^2^, area under the receiver operating characteristic curve (AUC), and F1-score to assess model fit and predictive performance. An AUC > 0.70 and F1-score ≤ 0.50 were accepted. P-values for the regression coefficients were determined using two-sided Wald tests.

## Results

### Demographics

**FIGURE 1:**
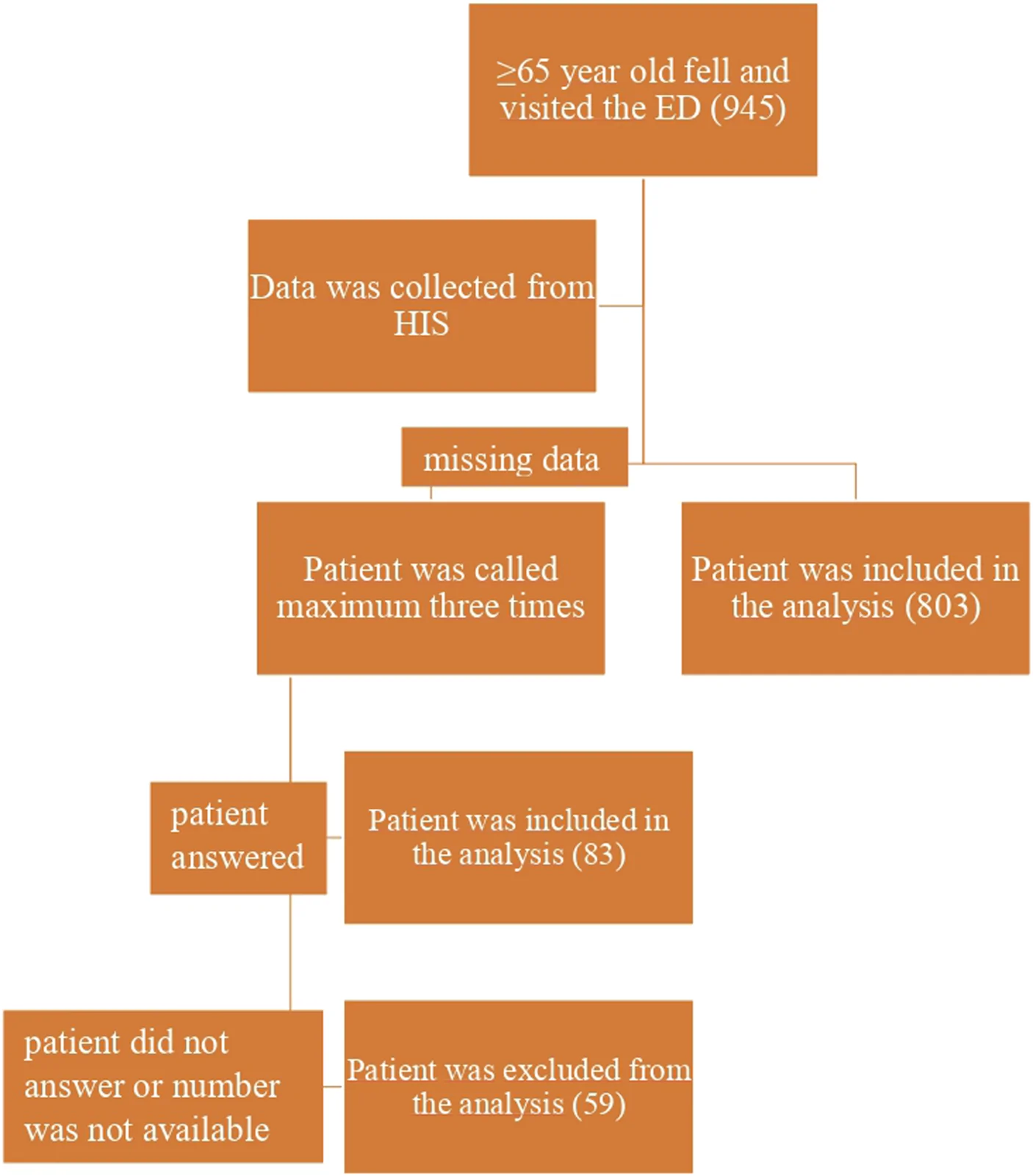
Flowchart of the collected data.

Between 21 March and 6 July 2022, 1,000 older adult were admitted to the ED due to falls, however there were older adults, who fell multiple times, after deleting these, we determined, that 945 elderly patients were admitted to the ED due to falls. We excluded 59 patients and collected data on the remaining 886, who were designated as the study group (Figure 1). From the three general practices, 1,364 elderly patients were enrolled as the control group. The baseline characteristics of the two groups are presented in Table 1. Blood sample was not collected in every case, only 332 patients from 886 in the study group and 194 patients of 1,364 patients in the control group. Due to the different and low number of laboratory tests, we did not include laboratory parameters further. In the study group, the proportions of women, older people aged >75 years, and patients with dementia and Alzheimer’s disease were significantly higher in the study group than in the control group (p < 0.01). Further information about the circumstances, time and consequences of falls are showed in Érszegi et al. (Érszegi and others et al., 2024)

**TABLE 1:** Baseline characteristics of the control (n = 1,364) and study (n = 886) groups.

Variables	Total patients n (%)	Study group n (%)	Control group n (%)	p-value
Incidence of fall				
No fall	1,364 (60.60)	0	1,364 (60.60)	​
Fall	886 (39.40)	886 (39.40)	0	​
Age in years				
65–75	1,154 (51.3)	307 (34.70)	847 (62.10)	​
>75	1,096 (48.7)	579 (65.30)	517 (37.90)	<0.01
Sex				
Male	811 (36.00)	268 (30.20)	543 (39.80)	​
Female	1,439 (64.00)	618 (69.80)	821 (60.20)	<0.01
Alzheimer				
No	2199 (97.7)	844 (95.30)	1,355 (99.30)	​
Yes	51 (2.3)	42 (4.70)	9 (0.70)	<0.01
Parkinson				
No	2178 (96.80)	856 (96.60)	1,322 (96.9)	​
Yes	72 (3.20)	30 (3.40)	42 (3.10)	0.69
Dementia				
No	2115 (94.00)	785 (88.60)	1,330 (97.50)	​
Yes	135 (6.00)	101 (11.40)	34 (2.50)	<0.01
Polypharmacy (≥5 drugs)				
No	838 (37.20)	288 (32.50)	550 (40.30)	​
Yes	1,412 (62.80)	598 (67.50)	814 (59.70)	<0.01

### Medications

The use of drugs on PIM lists was relatively high in both groups, with more than half of the general population using at least one EU (7)PIM, FORTA C/D, or PRISCUS-listed drug. The overall mean use of PIM was the highest when applying the EU (7)PIM list and the lowest with the FORTA C/D list. The use of PIM drugs was classified by sex, age, and neurodegenerative disease are presented in Tables 2–4.

**TABLE 2:** Use of potentially inappropriate medications by number in the study (n = 886) and control (n = 1,364) groups.

Use of PIM		Studied group n (%)	Control group n (%)	Overall n (%)
*EU7PIM*	Yes	664 (74.94)	888 (65.10)	1,552 (68.98)
	No	222 (25.06)	476 (34.90)	698 (31.02)
*FORTA C/D*	Yes	533 (60.16)	646 (47.36)	1,179 (52.40)
	No	353 (39.84)	718 (52.64)	1,071 (47.60)
*PRISCUS*	Yes	626 (70.65)	794 (58.21)	1,420 (63.11)
	No	260 (29.35)	570 (41.79)	830 (36.89)
No of PIM		n (%)	n (%)	n (%)
*EU7PIM*	1	206 (23.25)	340 (24.93)	546 (24.27)
	2	175 (19.75)	246 (18.04)	421 (18.71)
	3	149 (16.82)	165 (12.10)	314 (13.96)
	4	83 (9.37)	81 (5.94)	164 (7.29)
	5	35 (3.95)	29 (2.13)	64 (2.84)
*FORTA C/D*	1	245 (27.65)	350 (25.66)	595 (26.44)
	2	153 (17.27)	189 (13.86)	342 (15.20)
	3	90 (10.16)	74 (5.43)	164 (7.29)
	4	27 (3.05)	20 (1.47)	47 (2.09)
	5	14 (1.58)	11 (0.81)	25 (1.11)
*PRISCUS*	1	239 (26.96)	367 (26.91)	606 (26.94)
	2	187 (21.11)	244 (17.89)	431 (19.16)
	3	111 (12.53)	109 (7.99)	220 (9.78)
	4	51 (5.76)	51 (3.74)	102 (4.53)
	5	31 (3.50)	15 (1.10)	46 (2.04)
Mean and SD				
*EU7PIM*	​	1.82 ± 1.58	1.44 ± 1.51	1.59 ± 1.55
*FORTA C/D*		1.15 ± 1.26	0.80 ± 1.07	0.94 ± 1.16
*PRISCUS*		1.52 ± 1.42	1.11 ± 1.25	1.27 ± 1.34

**TABLE 3:** Use of potentially inappropriate medications by age group and sex (n = 2250).

Age groups n (%)				Gender n (%)
No of PIM	65–75 (n = 1,154)	75< (n = 1,096)	Male (n = 811)	Female (n = 1,439)
*EU(7)PIM*				
0	468 (40.55)	275 (25.09)	279 (34.40)	419 (29.12)
1	329 (28.51)	262 (23.91)	202 (24.91)	344 (23.91)
2	188 (16.29)	248 (22.63)	146 (18.00)	275 (19.11)
3	115 (9.97)	181 (16.51)	105 (12.95)	209 (14.52)
4	35 (3.03)	84 (7.66)	53 (6.54)	111 (7.71)
5≤	19 (1.65)	46 (4.20)	26 (3.21)	81 (5.63)
*FORTA C/D*				
0	652 (56.50)	419 (38.23)	429 (52.90)	642 (44.61)
1	272 (23.57)	323 (29.47)	204 (25.15)	391 (27.17)
2	146 (12.65)	196 (17.88)	111 (13.69)	231 (16.05)
3	55 (4.77)	109 (9.95)	45 (5.55)	119 (8.27)
4	19 (1.65)	28 (2.55)	11 (1.36)	36 (2.50)
5≤	10 (0.87)	21 (1.92)	11 (1.36)	20 (1.39)
*PRISCUS*				
0	524 (45.41)	306 (27.92)	324 (39.95)	507 (35.23)
1	321 (27.82)	285 (26.00)	222 (27.37)	383 (26.62)
2	172 (14.90)	259 (23.63)	150 (18.50)	283 (19.67)
3	84 (7.28)	136 (12.41)	69 (8.51)	150 (10.42)
4	37 (3.21)	65 (5.93)	34 (4.19)	67 (4.66)
5≤	16 (1.39)	45 (4.11)	12 (1.48)	49 (3.41)

**TABLE 4:** Use of potentially inappropriate medication by neurodegenerative disease (n = 2250).

​	Parkinson’s		Dementia n (%)		Alzheimer’s n (%)	
No of PIM	No (n = 2178) (%)	Yes (n = 72) (%)	No (n = 2115) (%)	Yes (n = 135) (%)	No (n = 2199) (%)	Yes (n = 51) (%)
*EU(7)PIM*						
0	687 (31.54)	11 (15.28)	675 (31.91)	23 (17.04)	689 (31.33)	9 (17.65)
1	529 (24.29)	17 (23.61)	520 (24.59)	26 (19.26)	534 (24.28)	12 (23.53)
2	405 (18.60)	16 (22.22)	397 (18.77)	24 (17.78)	412 (18.74)	9 (17.65)
3	302 (13.87)	12 (16.67)	282 (13.33)	32 (23.70)	304 (13.82)	10 (19.61)
4	154 (7.07)	10 (13.89)	147 (6.95)	17 (12.59)	156 (7.09)	8 (15.69)
5	101 (4.64)	6 (8.33)	94 (4.44)	13 (9.63)	104 (4.73)	3 (5.88)
*FORTA C/D*						
0	1,053 (48.35)	18 (25.00)	1,033 (48.84)	38 (28.15)	1,052 (47.84)	19 (37.25)
1	582 (26.72)	13 (18.06)	557 (26.34)	38 (28.15)	582 (26.47)	13 (25.49)
2	320 (14.69)	22 (30.56)	314 (14.85)	28 (20.74)	334 (15.19)	8 (15.69)
3	155 (7.12)	9 (12.50)	141 (6.67)	23 (17.04)	156 (7.09)	8 (15.69)
4	40 (1.84)	7 (9.72)	42 (1.99)	5 (3.70)	44 (2.00)	3 (5.88)
5	28 (1.29)	3 (4.17)	28 (1.32)	3 (2.22)	31 (1.41)	0 (0.00)
*PRISCUS*						
0	815 (37.42)	16 (22.22)	812 (38.39)	19 (14.07)	826 (37.56)	5 (9.80)
1	586 (26.90)	19 (26.39)	569 (26.90)	36 (26.67)	591 (26.88)	14 (27.45)
2	416 (19.10)	17 (23.61)	395 (18.68)	38 (28.15)	416 (18.92)	17 (33.33)
3	211 (9.69)	8 (11.11)	198 (9.36)	21 (15.56)	212 (9.64)	7 (13.73)
4	92 (4.22)	9 (12.50)	86 (4.07)	15 (11.11)	96 (4.37)	5 (9.80)
5	58 (2.66)	3 (4.17)	55 (2.60)	6 (4.44)	58 (2.64)	3 (5.88)

### Evaluated PIM lists in association with falling risk

For EU (7)PIM-listed drugs, in the full dataset, the use of 3 or 4 EU (7)PIM drugs was associated with a significantly increased falling risk, whereas in the adjusted subset, the use of 2–4 EU (7)PIM drugs was associated with a significantly higher risk of falls (Table 5).

**TABLE 5:** Use of European Union list of potentially inappropriate medications (EU [7]-PIM) in association with falls (full and adjusted datasets; adjusted for sex and neurodegenerative disease).

Variable	AOR	CI lower	CI upper	p value	Value_name	Value
Full dataset EU (7)PIM						
Alzheimer	3.718	1.731	8.724	0.001	R^2^	0.095
Dementia	2.617	1.692	4.122	0.000	AUC	0.701
Parkinson	0.736	0.437	1.224	0.242	F1 score	0.605
Gender	1.336	1.104	1.619	0.003	​	​
Age	1.078	1.065	1.092	0.000	​	​
Polypharmacy	0.849	0.658	1.094	0.206	​	​
EU (7)PIM 1	1.305	0.994	1.713	0.056	​	​
EU (7)PIM 2	1.363	0.991	1.874	0.057	​	​
EU (7)PIM 3	1.588	1.118	2.257	0.010	​	​
EU (7)PIM 4	1.793	1.173	2.741	0.007	​	​
EU (7)PIM 5	1.329	0.814	2.162	0.253	​	​
Balanced dataset EU (7)PIM						
Polypharmacy	1.110	0.855	1.439	0.433	R^2^	0.013
EU (7)PIM 1	1.201	0.907	1.591	0.200	AUC	0.577
EU (7)PIM 2	1.436	1.034	1.995	0.031	F1 score	0.643
EU (7)PIM 3	1.748	1.217	2.517	0.003	​	​
EU (7)PIM 4	2.136	1.369	3.356	0.001	​	​
EU (7)PIM 5	1.496	0.917	2.448	0.108	​	​

For FORTA C/D-listed drugs, the use of 1–5 FORTA C/D drugs in the full dataset and 1–4 FORTA C/D drugs in the adjusted subset was associated with a significantly increased falling risk (Table 6).

**TABLE 6:** Use of Fit fOR The Aged (FORTA C/D) drugs in association with falls (full and adjusted datasets).

Variable	AOR	CI lower	CI upper	p value	Value_name	Value
Full dataset FORTA C/D						
Alzheimer	3.811	1.776	8.939	0.001	R^2^	0.098
Dementia	2.549	1.647	4.019	0.000	AUC	0.704
Parkinson	0.681	0.401	1.141	0.149	F1 score	0.608
Gender	1.316	1.086	1.595	0.005	​	​
Age	1.077	1.064	1.091	0.000	​	​
Polypharmacy	0.874	0.697	1.095	0.241	​	​
FORTA C/D 1	1.273	1.001	1.617	0.049	​	​
FORTA C/D 2	1.379	1.027	1.850	0.033	​	​
FORTA C/D 3	1.844	1.255	2.712	0.002	​	​
FORTA C/D 4	2.067	1.083	3.988	0.028	​	​
FORTA C/D 5	2.443	1.138	5.372	0.023	​	​
Balanced dataset FORTA C/D						
Polypharmacy	1.150	0.915	1.446	0.229	R^2^	0.015
FORTA C/D 1	1.434	1.120	1.838	0.004	AUC	0.583
FORTA C/D 2	1.490	1.103	2.015	0.009	F1 score	0.632
FORTA C/D 3	2.112	1.431	3.141	0.000	​	​
FORTA C/D 4	2.183	1.140	4.328	0.021	​	​
FORTA C/D 5	1.984	0.925	4.444	0.084	​	​

For PRISCUS-listed drugs, the use of 1, 3, 4, or 5 PRISCUS drugs in the full dataset and 1–5 PRISCUS-listed drugs in the adjusted subset was associated with a significantly increased falling risk (Table 7).

**TABLE 7:** Use of PRISCUS drugs in association with falls (full and adjusted datasets; adjusted for sex and neurodegenerative disease).

Variable	AOR	CI lower	CI upper	p value	Value_name	Value
Full dataset PRISCUS						
Alzheimer	3.618	1.685	8.485	0.002	R^2^	0.098
Dementia	2.565	1.657	4.045	0.000	AUC	0.703
Parkinson	0.739	0.438	1.231	0.250	F1 score	0.603
Gender	1.326	1.095	1.608	0.004	​	​
Age	1.077	1.064	1.090	0.000	​	​
Polypharmacy	0.842	0.663	1.068	0.156	​	​
PRISCUS 1	1.339	1.038	1.728	0.025	​	​
PRISCUS 2	1.343	0.998	1.809	0.052	​	​
PRISCUS 3	1.857	1.297	2.660	0.001	​	​
PRISCUS 4	1.635	1.007	2.652	0.046	​	​
PRISCUS 5	2.458	1.359	4.515	0.003	​	​
Balanced dataset PRISCUS						
Polypharmacy	1.091	0.856	1.391	0.479	R^2^	0.016
PRISCUS 1	1.390	1.069	1.808	0.014	AUC	0.583
PRISCUS 2	1.557	1.148	2.114	0.004	F1 score	0.642
PRISCUS 3	2.045	1.408	2.982	0.000	​	​
PRISCUS 4	1.869	1.149	3.065	0.012	​	​
PRISCUS 5	2.638	1.464	4.895	0.002	​	​

**FIGURE 2:**
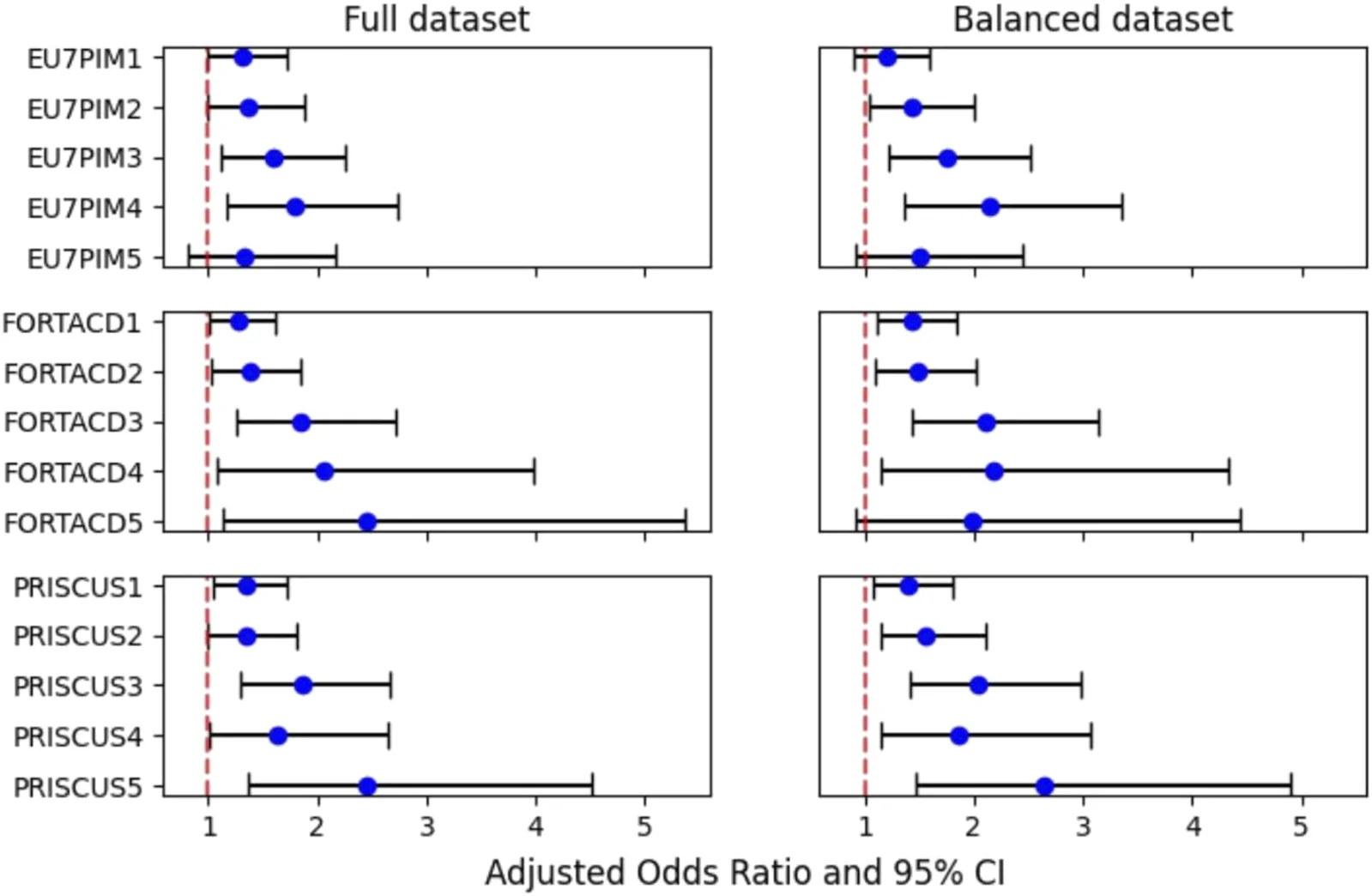
Full and adjusted datasets (adjusted odds ratios and 95% confidence intervals; adjusted for sex and neurodegenerative disease).

Forest plots for both the full and adjusted subsets are presented in Figure 2.

## Discussion

Previous studies have compared different PIM lists based on different end points, such as hospitalization risk (Heinz, 2016) or cognitive function (Krüger and others et al., 2021). The present study is the first to compare PIM lists based on falling risk. The rates of PIM-listed drug use for the three lists were high in both the control and study groups. In the study group, more than 25.00% of the patients used at least 1 PRISCUS- or FORTA C/D-listed drug, while more than 3.00% of the patients used 5 EU (7)PIM or PRISCUS-listed drugs. The literature distinguishes between appropriate and inappropriate polypharmacy; however, no consensus has been established for such categorization (Waldorff et al., 2023). However, in the present study, a new term, “polyPIMuse,” is introduced which is defined as the use of 5 or more PIM-listed drugs. In the polyPIMuse cases analyzed, the risk of falling increased significantly if the 5 PIM-listed drugs were listed in PRISCUS or FORTA C/D. However, in the latter case, increased fall risk was only significant in the full (not adjusted) dataset. PolyPIMuse of EU (7)PIM-listed drugs increased the risk of falling but not significantly in either dataset. Although our results indicate that the risk of falls increased with the number of PIM-listed drugs used, this association should be verified in a study with a larger dataset. The EU (7)PIM list is considered to be DOLA, the PRISCUS list is considered to be DOLA+, and the FORTA C/D list is considered to be PILA. Despite the differences between these lists, if the patient used at least one drug on one of these lists, their risk of falls increased. Previous studies have shown that certain drug classes (e.g., antipsychotics, antidepressants, benzodiazepines) increase the risk of falling. Our study shows that PIM lists, which help evaluate the suitability of medications for elderly patients, can also help assess the impact of these medications on fall risk in these patients. Such utility may benefit not only the patients but also the healthcare system. Thus, predictions can be used both as a basis for modifying medication and for assessing and managing fall risk of older individuals, which can benefit them, their families, and society through the reduction of healthcare costs associated with fall risk management and reduction due to medication use.

## Limitations

Our study did not record comorbidities other than Alzheimer’s disease, Parkinson’s disease, and dementia. Laboratory parameters (e.g., hematocrit and hemoglobin values) were not available or recorded. Furthermore, indications of the medications used were not recorded, although the FORTA list includes data on indications. Data for the control group were collected from general practice settings in accordance with the approved ethical protocol, which only permitted the collection of data on older adults who had fallen and been admitted to the ED. Consequently, controls represented community-dwelling older adults from the same source population who had not had a fall-related ED admission. Although the use of ED-based controls might have reduced differences in healthcare utilization between groups, this approach was considered appropriate for the predefined study population. Although we were able to obtain results for up to five PIM-listed drugs, cases of use of more than five PIM-listed drugs were fewer, which yielded less reliable results; thus, we excluded the analysis of such cases.

## Conclusion

Our study showed that the use of EU (7)PIM-, PRISCUS-, or FORTA C/D-listed drugs is associated with increased fall risk. Of the three lists, use of drugs on the PRISCUS list was most significantly associated with increased fall risk. Although the listing methods of these lists differ, our results indicate that the PIM lists can adequately predict the impact of the listed drugs on fall risk. In the future, more robust and more detailed studies (e.g., with all recorded comorbidities and laboratory parameters) with larger datasets should be conducted to verify the findings of the current study.

## Data Availability

The original contributions presented in the study are included in the article/supplementary material, further inquiries can be directed to the corresponding author.
